# Long‐Term Outcomes of Neoadjuvant Therapy Versus Upfront Surgery for Resectable Pancreatic Ductal Adenocarcinoma

**DOI:** 10.1002/cam4.70363

**Published:** 2024-11-17

**Authors:** Kyung In Shin, Min Sung Yoon, Jee Hoon Kim, Won Joon Jang, Galam Leem, Jung Hyun Jo, Moon Jae Chung, Jeong Youp Park, Seung Woo Park, Ho Kyoung Hwang, Chang Moo Kang, Seung‐seob Kim, Mi‐Suk Park, Hee Seung Lee, Seungmin Bang

**Affiliations:** ^1^ Division of Gastroenterology, Department of Internal Medicine Yonsei University College of Medicine Seoul Korea; ^2^ Institute of Gastroenterology Yonsei University College of Medicine Seoul Korea; ^3^ Department of Hepatobiliary and Pancreatic Surgery Yonsei University College of Medicine Seoul Korea; ^4^ Department of Radiology, Research Institute of Radiological Science Yonsei University College of Medicine Seoul Korea

**Keywords:** long‐term results, neoadjuvant therapy, overall survival, pancreatic neoplasm, progression‐free survival

## Abstract

**Introduction:**

This study aimed to compare the long‐term effects of neoadjuvant therapy and upfront surgery on overall survival (OS) and progression‐free survival (PFS) in patients with resectable pancreatic ductal adenocarcinoma (PDAC).

**Methods:**

We retrospectively analyzed 202 patients, including 167 who had upfront surgery and 35 who received neoadjuvant therapy followed by surgery. Surgical outcomes and survival rates were compared using propensity score matching to minimize selection bias.

**Results:**

Neoadjuvant therapy showed significantly longer 75% OS (72.7 months vs. 28.3 months, *p* = 0.032) and PFS (29.6 months vs. 13.2 months, *p* < 0.001) compared to upfront surgery. Additionally, neoadjuvant therapy demonstrated significant improvements in surgical outcomes, including higher R0 resection rates (74.3% vs. 49.5%, *p* = 0.034), reduced tumor size (22.0 mm vs. 28.0 mm, *p* = 0.001), and decreased lymphovascular invasion (20.0% vs. 52.4%, *p* = 0.001).

**Conclusion:**

Our study demonstrates the potential benefits of neoadjuvant therapy for resectable PDAC. The improved survival rates, delayed disease progression, and enhanced surgical outcomes underscore the potential of neoadjuvant therapy in addressing this aggressive disease. Despite limitations such as the retrospective design and small sample size, these findings support the effectiveness of neoadjuvant therapy in improving treatment outcomes for PDAC patients in real‐world settings. Further prospective studies are required to validate these results.

## Abstract 2

This study suggests that neoadjuvant therapy in patients with resectable pancreatic cancer improves overall survival and surgical outcomes, while also delaying disease progression compared to upfront surgery. These findings underscore the pivotal role of neoadjuvant therapy in treatment strategies for resectable pancreatic cancer.

## 1 Introduction

Pancreatic ductal adenocarcinoma (PDAC) is one of the highly aggressive types of cancer, with an overall 5‐year survival rate of approximately 10% [1, 2]. Only 20% of PDAC patients are eligible for curative surgical resection at the time of diagnosis, and within 2 years after curative resection, the recurrence rate reaches 80% [3, 4, 5, 6]. However, the rate of receiving adjuvant treatment after surgery is only 50% due to surgical complications and diminished performance status [7, 8, 9]. Consequently, neoadjuvant treatment has emerged as a critical strategy for reducing postoperative recurrence rates. However, the optimal approach remains a topic of ongoing debate, especially given the mixed results from recent studies [10, 11, 12].

Neoadjuvant therapy offers several potential advantages, such as eradicating micrometastases or microscopic invasion and reducing tumor size [13, 14]. These benefits can lead to an improved R0 resection rate, an important prognostic factor in PDAC [15, 16]. Additionally, it may help identify patients with rapidly progressive diseases, enabling them to avoid futile surgery [17, 18]. Additionally, neoadjuvant treatment allows the majority of patients to receive chemotherapy, even those who may not be eligible for further treatment after surgery [19].

We previously reported the initial results of the response to neoadjuvant therapy in patients with resectable PDAC, in comparison to the group that underwent upfront surgery [20]. At a median follow‐up of 23.0 months (range, 15.5–37.2 months) for patients in the upfront surgery group and 19.3 months (range, 6.7–29.6 months) for those in the neoadjuvant therapy group, we confirmed a significant improvement in surgical outcomes and progression‐free survival (PFS) within the neoadjuvant therapy group compared to the upfront surgery group (29.6 months vs. 15.1 months, *p* = 0.002). However, there was a limitation to the study due to the relatively short follow‐up period for patients, which posed a constraint in assessing survival benefits. Herein, we report the long‐term results of neoadjuvant therapy for resectable PDAC.

## 2 Materials and Methods

### 2.1 Study Population

This single‐center retrospective study included patients with resectable PDAC who underwent curative resection. We conducted a comprehensive review of the electronic medical records of all patients who underwent surgery with a curative intent for pancreatic cancer at Severance Hospital between January 2012 and August 2019 [20].

**FIGURE 1:**
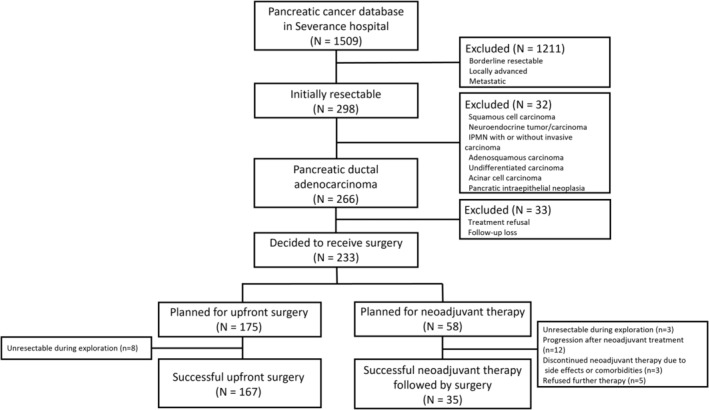
Patient diagram.

During this period, 1509 patients were registered in the Severance Hospital pancreatic cancer database. Among these, 298 patients diagnosed with radiologically resectable pancreatic cancer were included in this study. The exclusion criteria were as follows: (1) pathological findings indicating a diagnosis other than PDAC (*n* = 32) and (2) refusal of follow‐up treatment (*n* = 33). Of the remaining 233 patients, 175 were scheduled for upfront surgery, and eight patients from the upfront surgery group who were deemed unresectable during exploration were excluded. In the neoadjuvant group of 58 patients, the following cases were excluded: (1) inability to proceed with surgery due to PD during neoadjuvant therapy (*n* = 12); (2) patients who underwent surgery after neoadjuvant therapy but were deemed unresectable during exploration (*n* = 3); (3) treatment discontinuation due to side effects or comorbidities (*n* = 3); and (4) treatment refusal or discontinuation during neoadjuvant therapy (*n* = 5). Consequently, this study included 202 eligible patients who successfully underwent curative resection of resectable PDAC (Figure 1).

We categorized this cohort into two distinct groups: the upfront surgery cohort (*n* = 167, 82.7%) and the neoadjuvant treatment followed by the surgery cohort (*n* = 35, 17.3%). Upfront surgery was defined as cases in which preoperative chemotherapy or concurrent chemoradiotherapy (CCRT) was not administered, regardless of subsequent adjuvant therapy. Neoadjuvant therapy was defined as chemotherapy prior to surgery, with or without concurrent radiation.

The study was conducted in accordance with the ethical principles of the Declaration of Helsinki. Approval for this study was obtained from the Institutional Review Board of Severance Hospital (IRB number: 4‐2015‐1058). The need for informed consent was waived because of the retrospective nature of the study. The data that support the findings of this study are available on request from the corresponding author. The data are not publicly available due to privacy or ethical restrictions.

### 2.2 Preoperative Evaluation

Patients underwent a comprehensive preoperative assessment, including a clinical history, physical examination, laboratory tests, and radiologic assessment. Clinical data were retrieved from electronic medical records, including information such as age, sex, date of diagnosis, recurrence date, date of death, last follow‐up date, smoking and alcohol history, family history of pancreatic cancer, comorbidities, and tumor characteristics including location, clinical stage, and pathological stage. Additionally, details concerning antitumor treatments and surgical outcomes were collected.

Tumor resectability was assessed using three‐phase pancreatic computed tomography (CT) and abdominal magnetic resonance imaging (MRI), following the protocols outlined by the National Comprehensive Cancer Network (NCCN). Resectability was characterized by the absence of tumor contact with critical vessels such as the celiac axis, superior mesenteric artery, and common hepatic artery, as well as no involvement with the superior mesenteric or portal veins, or ≤ 180° contact without irregularity in vein contour. To assess resectability, all preoperative radiological images were thoroughly evaluated by two abdominal radiologists, both of whom were blinded to the surgical and pathological outcomes. The T, N, and M staging of tumors followed the guidelines outlined in the 8th edition of the American Joint Committee on Cancer staging system.

### 2.3 Outcomes

The primary outcome was overall survival (OS), which was defined as the duration until death from any underlying cause. Secondary endpoints included PFS and surgical outcomes, with particular emphasis on achieving margin‐negative (R0) resections. Resection was deemed microscopically complete (R0) if tumor cells were not detected within 1 mm of the resection margin, in line with the definition provided by the Royal College of Pathologists.

### 2.4 Statistical Analysis

Baseline characteristics were presented as either mean (standard deviation) or median (interquartile range) for continuous variables, while categorical variables were presented as numbers (percentages). Disparities between groups were assessed using the Student's *t*‐test or Mann–Whitney *U* test for continuous variables and the Fisher's exact test for categorical variables.

To reduce potential selection bias in the allocation of patients between the upfront surgery and neoadjuvant groups, 3:1 propensity score matching (PSM) was implemented. The propensity score models were adjusted for variables including age, sex, preoperative T stage, and preoperative N stage by employing the nearest‐neighbor matching approach. Statistical significance was established with a significance level of *p* < 0.05. PSM was executed using the MatchIt package within the R software.

We estimated the OS and PFS starting from the time of initial diagnosis for both the upfront surgery and neoadjuvant therapy groups. Kaplan–Meier curves were used to analyze OS and PFS in both matched and unmatched datasets. Survival analysis between the two groups was performed using the stratified log‐rank test. The association between the treatment modality and OS was evaluated using a Cox proportional hazards regression model. All statistical analyses were performed using R software, version 4.3.0, which was developed by The R Foundation in Vienna, Austria.

## 3 Results

### 3.1 Patients and Treatment

The baseline characteristics of the two groups are reported in Table 1. A total of 167 patients underwent upfront surgery, while 35 received neoadjuvant therapy for resectable PDAC. Among the 35 patients who underwent neoadjuvant treatment, 57% (*n* = 20) received neoadjuvant chemotherapy, and 43% (*n* = 15) underwent CCRT. FOLFIRINOX was the primary chemotherapeutic regimen (*n* = 17). In the upfront surgery group, 81.4% (*n* = 136) of the patients underwent adjuvant chemotherapy, whereas 85.7% (*n* = 30) of the patients in the neoadjuvant group received adjuvant chemotherapy. The specific regimens and their distributions are described in our previous publication [20].

**TABLE 1:** Comparative analysis of baseline characteristics between the upfront surgery and neoadjuvant therapy groups.

Variables	Unmatched			3:1 Matched		
	Upfront surgery (*n* = 167)	Neoadjuvant therapy (*n* = 35)	*p*	Upfront surgery (*n* = 105)	Neoadjuvant therapy (*n* = 35)	*p*
Demographic variables						
Age, year, median [IQR]	67.0 [58.0, 72.5]	60.0 [55.5, 69.5]	0.036	66.0 [55.0, 72.0]	60.0 [55.0, 69.5]	0.360
Age ≥ 70 years (%)	63 (37.7)	9 (25.7)	0.244	38 (36.2)	9 (25.7)	0.305
Female (%)	73 (43.7)	21 (60.0)	0.094	60 (57.1)	21 (60.0)	0.845
Tumor variables						
Tumor location (%)			0.435			0.800
Head	91 (54.5)	21 (60.0)		62 (59.0)	21 (60.0)	
Other	76 (45.5)	14 (40.0)		43 (41.0)	14 (40.0)	
Clinical T stage (%)			0.110			0.478
T1	54 (32.3)	7 (20.0)		26 (24.8)	7 (20.0)	
T2	103 (61.7)	23 (65.7)		71 (67.6)	23 (65.7)	
T3	10 (6.0)	5 (14.3)		8 (7.6)	5 (14.3)	
Clinical N stage (%)			0.030			0.375
N0	141 (84.4)	23 (65.7)		80 (76.2)	23 (65.7)	
N1	24 (14.4)	11 (31.4)		23 (21.9)	11 (31.4)	
N2	2 (1.2)	1 (2.9)		2 (1.9)	1 (2.9)	
CA 19–9, U/mL, median [IQR]	58.3 [18.9, 228.6]	64.2 [16.9, 402.3]	0.521	71.3 [20.5, 244.0]	64.2 [16.9, 402.3]	0.862
CA 19–9 ≥ 200 U/mL (%)	45 (26.9)	14 (40.0)	0.152	34 (32.4)	14 (40.0)	0.418
Operative method			0.342			0.580
PPPD	84 (50.3)	17 (48.6)		58 (55.2)	17 (48.6)	
Whipple's operation	2 (1.2)	2 (5.7)		2 (1.9)	2 (5.7)	
Distal pancreatectomy	73 (43.7)	15 (42.9)		41 (39.0)	15 (42.9)	
Total pancreatectomy	8 (4.8)	1 (2.9)		4 (3.8)	1 (2.9)	

Abbreviations: IQR, interquartile range; PPPD, pylorus preserving pancreaticoduodenectomy.

In terms of surgical procedures, among the 167 patients in the upfront surgery group, 50.3% (*n* = 84) underwent pylorus‐preserving pancreaticoduodenectomy (PPPD), 1.2% (*n* = 2) received Whipple's operation, 43.7% (*n* = 73) had distal pancreatectomy, and 4.8% (*n* = 8) underwent total pancreatectomy. In the neoadjuvant therapy group, 48.6% (*n* = 17) underwent PPPD, 5.7% (*n* = 2) underwent the Whipple operation, 42.9% (*n* = 15) underwent distal pancreatectomy, and 2.9% (*n* = 1) underwent total pancreatectomy.

To minimize selection bias, we conducted a 3:1 PSM matching based on age, sex, and clinical T and N stages. Following PSM, a cohort of 35 patients from the neoadjuvant therapy group was successfully matched with 105 patients who underwent upfront surgery. This matching process helped to create a more balanced comparison between the two groups in the study of resectable PDAC (Table 1).

### 3.2 Surgical Outcomes

As previously reported, the neoadjuvant therapy group showed statistically significant improvements in several surgical outcomes compared to the upfront surgery group, including a higher R0 resection rate (74.3% vs. 51.5%, *p* = 0.049), a smaller pathologic tumor size (22.0 mm vs. 27.0 mm, *p* = 0.004), an improved pathologic T stage (*p* = 0.026), and a reduced rate of lymphovascular invasion (20.0% vs. 40.7%, *p* = 0.022). After 3:1 PSM, the matched groups also demonstrated significant improvements in several factors, including a smaller pathologic tumor size (22.0 mm vs. 28.0 mm, *p* = 0.001), enhanced pathologic T stage (*p* = 0.009), a higher degree of histologic differentiation (*p* = 0.025), a reduced rate of lymphovascular invasion (20.0% vs. 52.4%, *p* = 0.001), and a higher R0 resection margin rate (74.3% vs. 49.5%, *p* = 0.034). Furthermore, differences in pathologic N stage (*p* = 0.031), the number of positive lymph nodes (0.74 vs. 1.66, *p* = 0.025), and positive lymph node/sampled lymph node ratio (5% vs. 11%, *p* = 0.038) were statistically significant between the two groups following a 3:1 PSM. A table showing surgical outcomes can be found in our previous publication [20].

### 3.3 Updated Survival Analysis

The median follow‐up duration for patients in the upfront surgery group was 30.7 months (range, 16.4–53.5 months), while for patients in the neoadjuvant therapy group, it was 50.5 months (range, 26.7–72.4 months).

**FIGURE 2:**
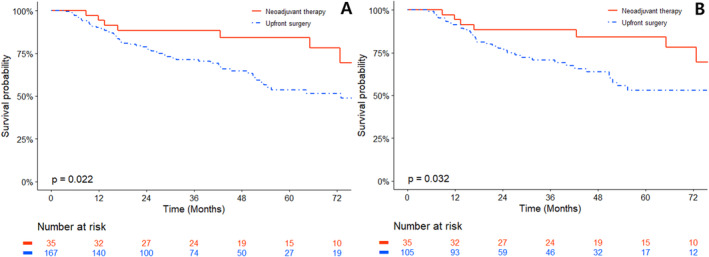
Kaplan–Meier plot of overall survival comparing neoadjuvant therapy to upfront surgery group. (A) Unmatched and (B) matched data are presented.

Throughout the follow‐up period, 66 patients died: 59 (35.3%) in the upfront surgery group and seven (20%) in the neoadjuvant therapy group. Compared to upfront surgery, neoadjuvant therapy demonstrated a more significant improvement in OS in both the unmatched (*p* = 0.022) (Figure 2A) and matched groups (*p* = 0.032) (Figure 2B). The Kaplan–Meier OS curves began to diverge at approximately 18 months. Instead of calculating the median OS, we computed the 75% OS because, at the conclusion of the follow‐up period, more than 50% of the patients in the neoadjuvant therapy group were still alive. The 75% OS in the matched groups was 28.3 and 72.7 months for patients in the upfront surgery and neoadjuvant therapy groups, respectively (Table 2). The highest separation point on the curves was observed at 60 months. The survival rates of the matched patients were 54.7% in the upfront surgery group and 84.2% in the neoadjuvant therapy group. The difference in survival rates at 5 years was statistically significant (HR, 0.34; 95% confidence interval (CI), 0.13–0.87; *p* = 0.018) (Table 3).

**TABLE 2:** Comparison of outcomes between upfront surgery and neoadjuvant therapy group.

	Unmatched			3:1 Matched		
Variables	Upfront surgery (*n* = 167)	Neoadjuvant therapy (*n* = 35)	*p*	Upfront surgery (*n* = 105)	Neoadjuvant therapy (*n* = 35)	*p*
Median OS, months (95% CI)	73.1 (52.8‐)	—	–—	80.5 (51.0‐)	—	—
Median PFS, months (95% CI)	15.2 (13.2–18.0)	29.6 (22.1‐)	0.002	13.2 (11.5–17.3)	29.6 (22.1‐)	< 0.001
75% OS, months (95% CI)	27.0 (18.2–42.7)	72.7 (42.5‐)	0.022	28.3 (17.5–50.8)	72.7 (42.5‐)	0.032
75% PFS, months (95% CI)	9.2 (7.6–10.7)	15.3 (9.5–23.3)	0.002	9.0 (7.0–10.6)	15.3 (9.5–23.3)	< 0.001
R0 resection rate, No. (%)	86 (51.5)	26 (74.3)	0.049	52 (49.5)	26 (74.3)	0.034

**TABLE 3:** Comparison of overall survival rates.

	Upfront surgery	Neoadjuvant therapy	Absolute difference (95% CI)	Hazard ratio (95% CI)	*p*
Unmatched					
Survival rate at 3 year (%)	71.3 (64.2–79.2)	88.2 (78.0–99.8)	16.9 (4.2–29.6)	0.40 (0.14–1.11)	0.067
Survival rate at 5 year (%)	53.6 (44.5–64.5)	84.2 (72.2–98.1)	30.6 (16.3–44.9)	0.33 (0.13–0.82)	0.012
3:1 Matched					
Survival rate at 3 year (%)	70.8 (61.7–81.2)	88.2 (78.0–99.8)	17.4 (3.6–31.2)	0.40 (0.14–1.14)	0.077
Survival rate at 5 year (%)	54.7 (43.6–68.7)	84.2 (72.2–98.1)	29.5 (14.1–44.9)	0.34 (0.13–0.87)	0.018

*Note:* Survival rates are estimated by Kaplan–Meier method, hazard ratios are calculated by Cox proportional hazard model, *p*‐values are estimated by log‐rank *p* test.

**FIGURE 3:**
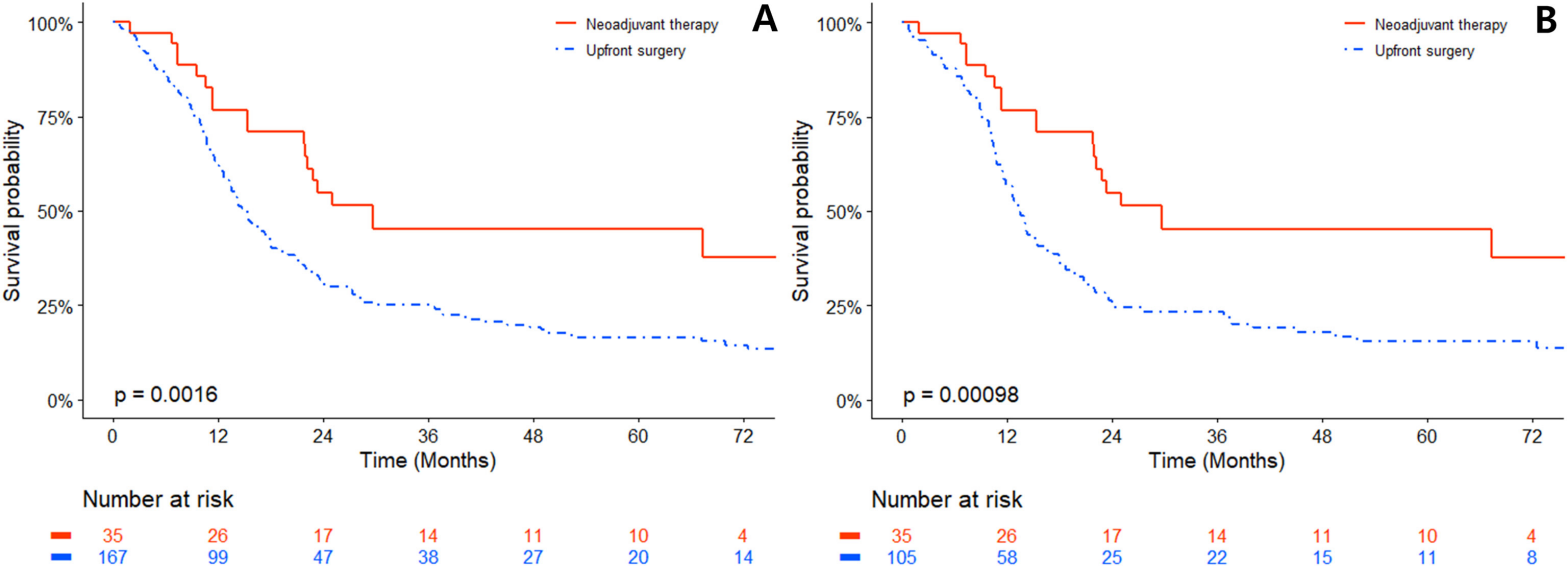
Kaplan–Meier plot of progression‐free survival comparing neoadjuvant therapy to upfront surgery group. (A) Unmatched and (B) matched data are presented.

During the follow‐up period, 81.4% (136/167) of the patients in the upfront surgery group and 54.3% (19/35) in the neoadjuvant therapy group experienced recurrence. During this timeframe, patients in the neoadjuvant therapy group demonstrated a significantly extended PFS compared with those in the upfront surgery group (Figure 3A,B). In the matched dataset, the median PFS was 13.2 months for the upfront surgery group and 29.6 months for the neoadjuvant therapy group (*p* < 0.001) (Table 2).

**FIGURE 4:**
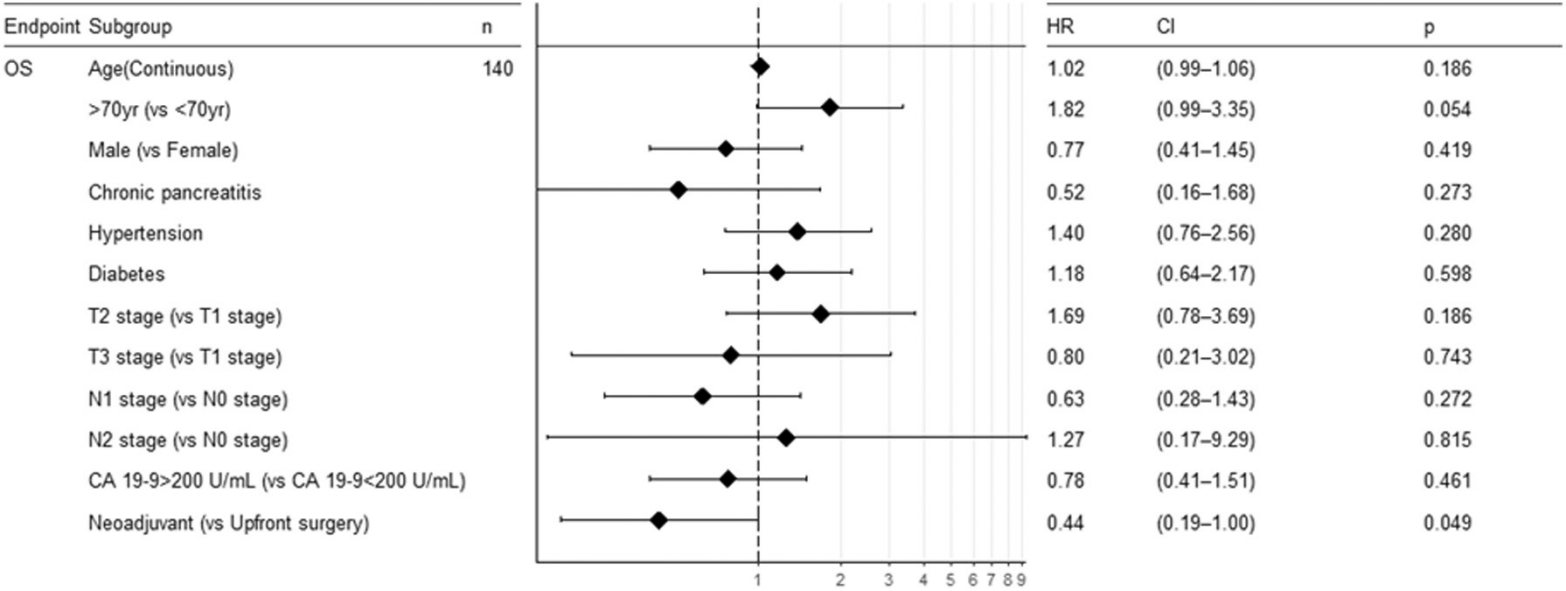
Multiple univariable Cox regression analysis of overall survival (OS).

**FIGURE 5:**
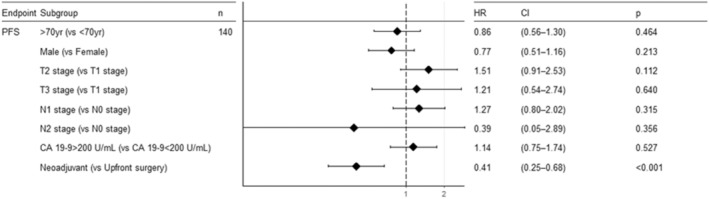
Multivariable Cox regression analysis of progression‐free survival (PFS).

In the univariable Cox analysis for OS, no statistically significant results were observed except for neoadjuvant therapy itself (HR, 0.43; 95% CI, 0.19 to 0.98; *p* = 0.045) (Figure 4). In the univariate analysis of PFS, significant prognostic factors were identified, including alcohol history, tumor location, and use of neoadjuvant therapy. After adjusting for confounding factors, the statistically significant effect of neoadjuvant therapy on PFS was observed (HR, 0.41; 95% CI, 0.24 to 0.68; *p* < 0.001) (Figure 5).

### 3.4 Intention‐To‐Treat Analysis

An intention‐to‐treat (ITT) analysis was performed to include all patients initially allocated to the neoadjuvant and upfront surgery groups, regardless of whether they ultimately underwent resection In this analysis, 58 patients were included in the neoadjuvant group and 175 patients in the upfront surgery group (Figure 1). The median PFS was 15.0 months in the neoadjuvant group and 14.2 months in the upfront surgery group (*p* = 0.5). And 75% OS was 26.5 months in the neoadjuvant group and 16.7 months in the upfront surgery group (*p* = 0.8). Even after 1:2 PSM analysis, no significant differences were observed in OS and PFS (*p* = 0.71 and *p* = 0.7, respectively) (Table S1).

## 4 Discussion

This updated analysis revealed a substantial long‐term survival advantage associated with neoadjuvant therapy compared with upfront surgery in patients diagnosed with resectable PDAC (HR, 0.43; *p* = 0.045). The 5‐year OS rate demonstrated a substantial and clinically significant improvement of 30.6% (HR, 0.33; *p* = 0.012), highlighting its potential as a promising treatment strategy for this aggressive malignancy. PFS and surgical outcomes, including the R0 resection rate, were also significantly superior to those in the upfront surgery group. The observed improvements in PFS further underscore the impact of neoadjuvant therapy on disease control, indicating its potential to delay disease recurrence and progression (HR, 0.41; *p* < 0.001). Statistically significant findings related to PFS observed in earlier studies have consistently maintained significance, even after a prolonged follow‐up period.

In the initial results of our study, OS did not exhibit a statistically significant difference between the two groups (*p* = 0.290) after a median follow‐up of 23.0 and 19.3 months, respectively [20]. At the time of the current analysis, the median follow‐up duration was 30.7 months for patients in the upfront surgery group and 50.5 months for patients in the neoadjuvant therapy group (Figure S1). Other studies comparing perioperative therapy in pancreatic cancer have suggested that a substantial follow‐up period is necessary to identify meaningful differences in survival [4, 12]. For instance, the PREOPANC trial, which contrasted neoadjuvant chemoradiotherapy with upfront surgery for resectable and borderline resectable pancreatic cancer, initially did not observe a survival discrepancy [21]. However, over an extended follow‐up duration, a notable enhancement in OS was indeed confirmed (HR, 0.73; 95% CI, 0.56 to 0.96; *p* = 0.025) [12]. In the present study, the survival curve initially exhibited a comparable rate of change and started to clearly divide around 18 months from diagnosis, resulting in a survival rate difference of 17.4% at 3 years and 29.5% at 5 years. This implies that prolonged follow‐up is necessary to reveal clinically significant survival disparities in neoadjuvant studies for PDAC.

Over the past decade, there has been a notable increase in the utilization of neoadjuvant systemic therapy for PDAC, sometimes accompanied by radiation [13, 22]. The NCCN guidelines also recommend neoadjuvant therapy as one of the two treatment options for resectable PDAC [23]. Nonetheless, there is still a lack of substantial research evidence to support this. Although certain studies have demonstrated that neoadjuvant therapy is more effective than upfront surgery in resectable PDAC, these findings are compromised because of the lack of an accurate assessment of PDAC resectability or the utilization of treatment regimens that are currently not commonly employed [12, 15, 22, 24]. In real‐world clinical practice, determining resectability plays a pivotal role in ascertaining the viability of resection for PDAC [13]. In our study, two experienced radiologists independently conducted preoperative imaging evaluations according to the NCCN guidelines, separate from surgical and pathological results. To the best of our knowledge, this is the first study to explore the potential advantages of neoadjuvant therapy, specifically involving FOLFIRINOX, for patients with resectable PDAC, excluding ongoing trials.

The recent multicenter randomized phase 2 trial by Labori et al. did not demonstrate a survival benefit from neoadjuvant FOLFIRINOX in patients with resectable PDAC when compared with upfront surgery [10]. However, there are still important considerations regarding the efficacy of neoadjuvant therapy. In the NORPACT‐1 trial, [4] the duration of neoadjuvant chemotherapy was relatively short at 2 months, and only 52% of patients in the experimental group completed the neoadjuvant chemotherapy. Moreover, fewer patients in the neoadjuvant group received adjuvant chemotherapy postsurgery (66% vs. 75%), and the proportion of patients receiving FOLFIRINOX as adjuvant therapy was lower in the neoadjuvant group compared to the upfront surgery group (25% vs. 43%). These imbalances must be considered when interpreting the trial results. Despite these limitations, it is notable that the R0 and N0 resection rates were higher in the neoadjuvant group, which suggests that long‐term results may provide further insights into the potential benefits of neoadjuvant therapy. Future research is needed to determine the optimal duration of neoadjuvant chemotherapy and the type of adjuvant chemotherapy postsurgery.

In resectable PDAC, one of the major challenges of neoadjuvant therapy is the potential delay or inability to proceed with surgery due to disease progression or adverse effects during treatment. This issue can also diminish the overall outcomes of neoadjuvant therapy. Following our previous study, additional chart reviews were conducted for the ITT analysis. In this analysis, we included all patients initially assigned to the neoadjuvant therapy and upfront surgery groups, regardless of whether they ultimately underwent resection. This approach did not reveal significant differences in survival and disease progression between the two groups. While ITT analysis plays a role in reducing selection bias and providing a comprehensive view of treatment efficacy, it is important to interpret these results considering the inherent challenges and potential drawbacks of neoadjuvant therapy. Given that surgery remains the standard of care for resectable pancreatic cancer, neoadjuvant treatment is typically selected only in special cases, making ITT analysis challenging in real‐world settings, which is also a limitation of retrospective studies. Other retrospective studies on neoadjuvant therapy in pancreatic cancer have also focused on patients who proceeded to surgery after neoadjuvant treatment [15, 25]. Our study was designed with the same methodology as our previous research to observe the long‐term data, aiming to assess the real‐world clinical outcomes of neoadjuvant therapy.

Of the 58 patients who received neoadjuvant therapy, 35 (60%) were able to proceed with surgery, which aligns with resectability rates reported in previous studies, ranging from 55% to 86% [10, 11, 26]. Among the patients who were unable to undergo surgery, seven experienced distant metastases within 2 months, suggesting the possibility of occult metastasis. This finding implies that early metastasis can be anticipated if they undergo upfront surgery. This highlights neoadjuvant therapy's potential to detect occult metastasis, preventing unnecessary surgery. Additionally, in a recent retrospective study on borderline resectable PDAC, it was reported that there was no significant difference in OS between cases where neoadjuvant chemotherapy was administered but surgery could not proceed and cases where upfront surgery was performed [27].

The objective of our study was not to present a definitive answer for resectable PDAC but rather to explore the potential significance of neoadjuvant therapy through real‐world data, considering the possibility of micrometastasis at diagnosis and the high rate of early recurrence in pancreatic cancer. There is still insufficient evidence to determine which patients should be selected for a neoadjuvant strategy. Determining the appropriateness of neoadjuvant therapy cannot rely solely on imaging studies, such as CT or MRI, as assessing primary lesions alone may be insufficient [28, 29]. Previous studies have reported the importance of factors beyond imaging, including germline mutations, tumor subtype, CA 19–9 levels, patient performance status, excessive weight loss, and other clinical parameters [30, 31, 32]. Therefore, there is a need for the development of biomarkers or biochemical parameters to effectively evaluate patients suitable for neoadjuvant therapy.

Our study supports the benefits of neoadjuvant therapy but has a few limitations to consider. First, this was a retrospective study from a single center, which may have introduced bias and limited generalizability. To mitigate selection bias, we performed PSM for clinical variables that exhibited disparities between the two groups, thereby increasing the reliability of our comparison. Second, since this study builds upon the long‐term data of a previous study, the research design primarily focused on a per‐protocol analysis, which included patients who successfully proceeded to surgery. The ITT analysis did not demonstrate a significant improvement in survival, which limits the interpretation of our findings. The primary aim of this study is to demonstrate, using real‐world data, that neoadjuvant therapy can enhance both surgical and survival outcomes in patients with resectable pancreatic cancer, underscoring the importance of well‐designed prospective studies. Third, the disparity in median follow‐up duration could potentially introduce bias into observed outcomes. However, as shown in the KM curve, the gap continues to widen after 18 months, and as seen in Figure S1, long‐term survival is confirmed in patients without recurrence. In this study, the proportion of long‐term survivors over 3 years was 68.6% (24 of 35) in the neoadjuvant group and 44.3% (74 of 167) in the upfront surgery group. Therefore, it is expected that differences in survival will become more pronounced with longer follow‐up periods. Future prospective studies should aim to compare outcomes with identical follow‐up periods to provide a more accurate assessment. Finally, the neoadjuvant therapy group received a range of treatment regimens, such as chemotherapy and chemoradiotherapy, potentially leading to variability in outcomes owing to differing treatment responses. Future prospective studies will require sizable, homogeneous samples and standardized protocols for a comprehensive analysis.

## 5 Conclusion

Our study demonstrates the potential benefits of neoadjuvant therapy for resectable PDAC. The improved surgical outcomes, delayed disease progression, and extended survival underscore the promise of this treatment approach. Additionally, the study identified cases with a potential for early recurrence. Despite the limitations of being a retrospective, single‐center study, our research shows the effectiveness of neoadjuvant treatment in resectable PDAC in real‐world data. Future research should prioritize prospective multicenter studies and consider perioperative treatment protocols, optimal patient selection for neoadjuvant therapy, and the development of biomarkers to predict treatment response.

## Author Contributions

**Kyung In Shin:** conceptualization (equal), funding acquisition (equal), writing – original draft (lead). **Min Sung Yoon:** data curation (lead), methodology (equal), writing – review and editing (equal). **Jee Hoon Kim:** formal analysis (equal), investigation (equal). **Won Joon Jang:** data curation (equal), investigation (equal). **Galam Leem:** data curation (equal), investigation (equal). **Jung Hyun Jo:** investigation (equal), supervision (equal). **Moon Jae Chung:** resources (equal), writing – review and editing (equal). **Jeong Youp Park:** resources (equal), supervision (equal). **Seung Woo Park:** supervision (equal), writing – review and editing (equal). **Ho Kyoung Hwang:** resources (equal). **Chang Moo Kang:** conceptualization (equal), resources (equal). **Seung‐seob Kim:** resources (equal). **Mi‐Suk Park:** resources (equal), validation (equal). **Hee Seung Lee:** conceptualization (equal), project administration (equal), writing – review and editing (equal). **Seungmin Bang:** conceptualization (equal), project administration (lead), supervision (equal), writing – review and editing (lead).

## Ethics Statement

Approval from the Institutional Review Board of Severance Hospital (IRB number: 4‐2015‐1058).

## Consent

The need for informed consent was waived because of the retrospective nature of the study.

## Conflicts of Interest

The authors declare no conflicts of interest.

## Supporting information


Figure S1.

Table S1.

## Data Availability

The data that support the findings of this study are available on request from the corresponding author. The data are not publicly available due to privacy or ethical restrictions.
