# Supplementary figures and images for: Long‐Term Outcomes of Neoadjuvant Therapy Versus Upfront Surgery for Resectable Pancreatic Ductal Adenocarcinoma
Source: Cancer Med. 2024 Nov 17;13(22):e70363. doi: 10.1002/cam4.70363 (PMC11570550; doi:10.1002/cam4.70363)

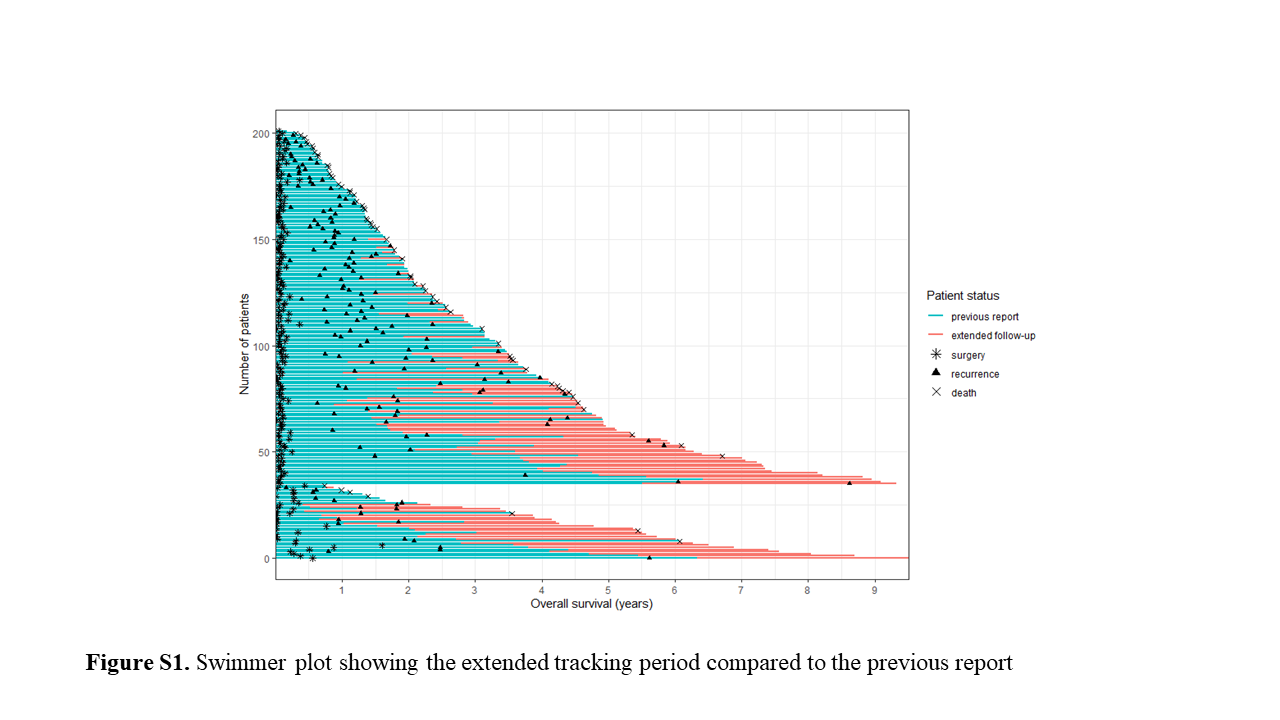

Supplement: Supplementary file 1 — Figure S1. [file CAM4-13-e70363-s001.tif]
