# Supplementary material for: Long‐Term Outcomes of Neoadjuvant Therapy Versus Upfront Surgery for Resectable Pancreatic Ductal Adenocarcinoma
Source: Cancer Med. 2024 Nov 17;13(22):e70363. doi: 10.1002/cam4.70363 (PMC11570550; doi:10.1002/cam4.70363)
Supplement: Supplementary file 2 — Table S1. [file CAM4-13-e70363-s002.docx]

| **Table S1.** Comparison of survival outcomes of ITT analysis between upfront surgery and neoadjuvant therapy group | | | | | | | | | | | | | |
| --- | --- | --- | --- | --- | --- | --- | --- | --- | --- | --- | --- | --- | --- |
|  |  |  | Unmatched | | | | |  | 2:1 Matched | | | | |
|  |  |  | Upfront surgery | | Neoadjuvant therapy | |  |  | Upfront surgery | | Neoadjuvant therapy | |  |
| Variables | |  | (n= 175) | | (n= 58) | | *P* value |  | (n= 116) | | (n= 58) | | *P* value |
| Median OS, months (95% CI) | | | 73.1 (52.8-NR) | | NR | | - |  | 80.5 (50.8-NR) | | NR | | - |
| Median PFS, months (95% CI) | | | 14.2 (12.6-17.4) | | 15.0 (10.6-23.3) | | 0.500 |  | 13.4 (11.8-17.3) | | 15.0 (10.6-23.3) | | 0.700 |
| 75% OS, months (95% CI) | | | 26.5 (18.2-42.4) | | 16.7 (11.8-26.5) | | 0.800 |  | 22.8 (16.0-42.2) | | 16.7 (11.8-NR) | | 0.700 |
| 75% PFS, months (95% CI) | | | 8.9 (7.0-10.6) | | 3.6 (2.8-10.6) | | 0.500 |  | 3.6 (2.8-10.6) | | 8.9 (6.9-10.6) | | 0.700 |
| NR, not reached | |  |  |  |  |  |  |  |  |  |  |  |  |
